# Pharmacodynamic Parameters of Pharmacokinetic/Pharmacodynamic (PK/PD) Integration Models

**DOI:** 10.3389/fvets.2022.860472

**Published:** 2022-03-24

**Authors:** Longfei Zhang, Hongbing Xie, Yongqiang Wang, Hongjuan Wang, Jianhe Hu, Gaiping Zhang

**Affiliations:** ^1^Postdoctoral Research Station, Henan Agriculture University, Zhengzhou, China; ^2^College of Animal Science and Veterinary Medicine, Henan Institute of Science and Technology, Xinxiang, China; ^3^Postdoctoral Research Base, Henan Institute of Science and Technology, Xinxiang, China

**Keywords:** kill rate, mutant prevention concentration, PK/PD integration model, PD parameter, time-kill curve, MIC, multi-drug resistance, dosage regimen

## Abstract

Pharmacokinetic/pharmacodynamic (PK/PD) integration models are used to investigate the antimicrobial activity characteristics of drugs targeting pathogenic bacteria through comprehensive analysis of the interactions between PK and PD parameters. PK/PD models have been widely applied in the development of new drugs, optimization of the dosage regimen, and prevention and treatment of drug-resistant bacteria. In PK/PD analysis, minimal inhibitory concentration (MIC) is the most commonly applied PD parameter. However, accurately determining MIC is challenging and this can influence the therapeutic effect. Therefore, it is necessary to optimize PD indices to generate more rational results. Researchers have attempted to optimize PD parameters using mutant prevention concentration (MPC)-based PK/PD models, multiple PD parameter-based PK/PD models, kill rate-based PK/PD models, and others. In this review, we discuss progress on PD parameters for PK/PD models to provide a valuable reference for drug development, determining the dosage regimen, and preventing drug-resistant mutations.

## Introduction

The excessive use of antibiotics has promoted the emergence and selection of multi-drug-resistant (MDR) pathogenic bacteria. MDR cause infections that threaten the health of humans, livestock, and wild animals. Some Gram-negative (G^−^) bacteria harboring the mobilized colistin resistance (*mcr*) gene are resistant to colistin, and this is leading to fewer and fewer antibiotics available for clinical treatment of MDR infections (1, 2). Indeed, in the future there may be no drugs available to treat bacterial infections caused by MDR pathogens. The main approaches to address this include the development of new drugs, optimization of dosage regimen, revision of susceptible breakpoints, and drug combinations. Pharmacokinetic/pharmacodynamic (PK/PD) models can comprehensively investigate interactions between hosts, pathogens, and drugs, and the obtained PK/PD parameters can predict the clinical antibacterial efficacy (3, 4). Thus, PK/PD integration is an effective and practical method for addressing MDR bacterial infections, and it has been widely applied to optimize the dosage regimen and reset the bacterial susceptible breakpoints. Theoretically, the appropriate application of PK/PD integration has potential to improve the outcomes on recovering antimicrobial activity, extending the usage life of an antimicrobial, and preventing the emergence and spread of resistant bacteria (5–9).

In PK/PD integration models, the most commonly applied PK/PD parameters are based on minimal inhibitory concentration (MIC). MIC is a standard *in vitro* value for a given antibacterial against a specific microorganism after a fixed time period (16–18 h). In other words, MIC is a net result when the growth rate of microorganisms (the population of inoculum) equal to the kill rate of a given drug at the fixed concentration which different from the dynamic character of drug concentrations *in vivo*. So, MIC is a hybrid and contextual PD variable due to its nature and the conditions for obtaining it (10, 11). The test medium play an important influence on the numerical value of a MIC because of a possible large matrix effect. In standard test, the Mueller Hinton Broth (MHB) was chosen because it ensures the rapid growth of bacteria. But the growth rate can be much slower *in vivo* which will inevitably change the value of the MIC because the difference of the growth rate, the duration of the incubations, and the size of the starting inoculum. For example, the macrolides have a large difference between *in vitro* MIC and effective *in vivo* plasma concentrations because of a very significant matrix effect which has been demonstrated for all veterinary macrolides and well explained by the fact that the MHB promotes the overexpression of efflux pump (12). The test method of MIC may also result in treatment failure and emergence of resistant mutations in bacterial infections (13–15). (a) The determination of MIC is an all-or-nothing result; a drug will exhibit antibacterial activity when its concentration is above the MIC, and have no antibacterial effect when its concentration is below the MIC. This does not reflect the real antibacterial activity of low drug concentrations against pathogens because different types of drugs have different antibacterial characteristics based on their antibacterial mechanisms. (b) MIC is typically determined using the double dilution method, which may result in estimated values that are higher than real values. Administration of a dosage regimen based on MIC may result in drug residues and ecological damage after a long time. (c) MIC is determined using static drug concentrations, which does not reflect the dynamic antibacterial activities of drugs against pathogens, such as changes in kill rate and growth rate in the presence of different drug concentrations over different time periods. (d) The bacterial cell density is typically ~10^5^ CFU/mL for MIC determination. Although this density not representative of *in vivo* infectious situations (the bacterial number may exceed 10^8^ CFU/mL in serious infection), the mutant frequency (the natural mutant occurrence frequency is ~10^−6^) may largely increase when amount of bacteria is exposed to antibacterial drugs, such as fluoroquinolones. These limitations may result in the emergence of MDR bacteria. Thus, developing new drugs and designing dosage regimens based only on MIC for PK/PD simulation is not sufficient. Therefore, it is necessary to explore PD parameters to more accurately investigate the antibacterial activities of drugs against pathogens.

In order to overcome the limitations of MIC-based PK/PD integration in clinical treatment, researchers have conducted numerous studies to optimize PD parameters, including mutant prevention concentration (MPC)-based PK/PD models, multiple PD parameter-based PK/PD models, and kill rate-based PK/PD models. Therefore, this review firstly covers the basic methods, concepts, and mathematical models of MIC-based PK/PD integration. And then introduces other PD parameter-based PK/PD integration approaches. This work provides a valuable guidance for optimizing the dosage regimen, developing new drugs, and preventing the emergence of MDR bacteria.

## Basic Methods of PK/PD Integration

PK/PD integration models include *in vitro, ex vivo*, and *in vivo* PK/PD models. For *in vitro* PK/PD studies, peristaltic pump and hollow fiber models are the most commonly used methods for simulating PK processes of drugs in hosts. The peristaltic pump model was applied to simulate one-compartment, two-compartment model, and multiple-compartment models. The simplest peristaltic pump model consists of a storage compartment (blank medium), a central compartment (drug, bacteria, and medium), and an elimination compartment (waste medium) connected by rubber pipe, and the PK characteristics (elimination rate) of drugs in hosts are simulated by a peristaltic pump. A magnetic bar is applied to mix the medium, and a thermostat is used to ensure the optimal growth temperature for pathogens. After measuring the drug concentration and bacterial cell density, the antibacterial effect and PK/PD parameters are calculated and simulated to guide the design of the dosage regimen (16–21). The hollow fiber model consists of thousands of hollow fiber tubes that simulate multiple compartments (22–29). The *in vitro* PK/PD model is simple, economical, and easy to operate, and it can directly describe the dynamic interaction between drugs and pathogens.

The *ex vivo* PK/PD model is used to investigate the antibacterial activities of hosts, drugs, and bacteria in drug-containing body fluids rather than artificial medium. The most commonly applied body fluids are plasma, serum, and tissue cage fluid (TCF). After collecting these samples at different timepoints following drug administration, drug concentrations and time-kill curves are determined, and a mathematical equation is applied to analyze the relationships between PK/PD parameters and antibacterial effect. TCF is a type of extracellular fluid that is typically targeted during bacterial infection. Thus, TCF is an ideal medium for *ex vivo* PK/PD integration studies. TCF can be acquired through surgery by implanting a tissue cage between muscle and skin. After ~4 weeks, granulation tissue surrounds the tissue cage and produces TCF. The TCF model has been widely applied in pig, rabbit, cattle, camel, goat, and other animals (30–39). Uterine fluid (40) and intestinal juice (41) have also been used for *ex vivo* PK/PD studies. However, although drug concentrations are still static when study *ex vivo* kill curves, this model can enable sampling of the extracellular fluid at different times after dosing which allow collection of samples as the concentration changes and partially consider the influence of the host.

Previous *in vivo* PK/PD models have been used to study interactions between hosts, drugs, and pathogens. The most commonly used are the tissue cage infection model (TCIM) and the target organ infection model (TOIM). The TCIM was established by adding pathogens to the tissue cage model, a series of dosage regimens were subsequently administrated for treatment, and TCF samples were collected for drug concentration measurement and bacterial population counting at different timepoints. After calculating the PK/PD parameters and antibacterial effect (the change in bacterial population), the relationship between PK/PD parameters and antibacterial effect was fitted and the dosage was predicted (42–49). The TOIM approach can simulate clinical infection by directly inoculating bacteria into target organs, then administering drugs at different dosages and intervals. Finally, animals are killed at different timepoints for bacterial population counting and antibacterial concentration determination at infected sites. The obtained PK/PD parameters and antibacterial effect are then analyzed and evaluated. These *in vivo* PK/PD models can comprehensively study the interactions between hosts, drugs, and pathogenic bacteria, and the results are typically consistent with clinical treatment (50–62).

## Basic PK/PD Parameters and Mathematical Models

**Figure 1:**
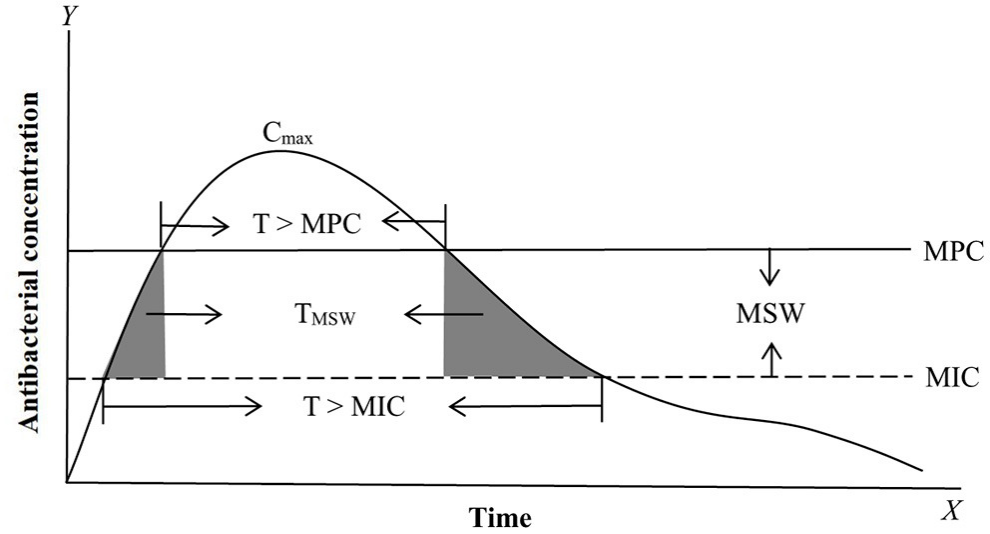
Antimicrobial PK, PD, and PK/PD parameters based on MIC (minimum inhibitory concentration) and MPC (mutant prevention concentration). The most commonly applied PK/PD parameters are AUC/MIC (area under the curve of antibacterial concentration divide MIC) or AUC/MPC, C_max_/MIC (maximum drug concentration divide MIC) or C_max_/MPC, and T > MIC (the time of the drug concentration above MIC during the dosage interval) or T > MPC (the time of the drug concentration above MPC during the dosage interval). MSW (mutant selection window) is the drug concentration between MIC and MPC. T_MSW_ (dash area) is the time that the antibacterial concentration inside MSW during the dosage interval.

For PK/PD integration, MIC is the most commonly applied PD variable used to calculate PK/PD indices such as AUC/MIC (area under the curve of concentration divided by MIC), C_max_/MIC (maximum concentration divided by MIC), and %T >MIC (percentage of time that drug concentrations exceed MIC during the dosage interval) (50, 63–67) (Figure 1). Actually, all PK/PD indices must be calculated with free concentrations and not with total plasma concentrations because MIC is a free concentration. Based on the closeness of the relationship between PK/PD parameters and antibacterial effect, antibacterial drugs can generally be divided into time-dependent drugs, concentration-dependent, and co-dependent drugs (3, 68–70). The represented PK/PD parameter for time-dependent drugs is %T >MIC. This reflects an antibacterial effect that is closely related to the duration of the drug concentration above MIC; the longer the duration of the drug concentration above MIC, the better the antibacterial activity. Time-dependent drugs include β-lactam antibiotics and macrolides. However, different members of macrolides have different PK/PD dependencies (71), so, the antibacterial activity should be evaluated prudently. For concentration-dependent drugs, representative PK/PD parameters are AUC/MIC and C_max_/MIC. These reflect antibacterial effects that are closely related to drug concentrations above MIC; the higher of drug concentration, the better of the antibacterial activity. Well-known concentration-dependent drugs are aminoglycosides and fluoroquinolones. However, for aminoglycosides, the more appropriate PK/PD parameter is AUC/MIC rather than C_max_/MIC, because the C_max_/MIC criterion was historically proposed for rodents and can not reflect the over time process (72, 73). For co-dependent drugs, the PK/PD parameter were AUC/MIC or %T >MIC against different bacteria, such as tetracycline and glycopeptides.

To confirm the most appropriate PK/PD parameters and predict the values of PK/PD parameters for designing the dosage regimen, a mathematical model is needed. A commonly applied model for this purpose is the Sigmoid E_max_ model (Equation 1). This sigmoid concentration-response model is a strongly empirical model. This model is a useful tool to formalize our understanding of experimental data, but it has no clear relationship to real physical characteristics of the actual system being studied.


(1) $$\begin{array}{l} \text{E}=\frac{{\text{E}}_{\text{max}}\times {\text{C}}^{\text{N}}}{\text{E}{\text{C}}_{50}+{\text{C}}^{\text{N}}} \end{array}$$

where E_max_ is the maximum antibacterial effect of a drug, EC_50_ is the value of PK/PD parameters or drug concentrations at 50% E_max_, C is the antibacterial concentration or PK/PD parameter, and N (Hill coefficient) is the slope of the fitted curves between PK/PD parameters or drug concentrations and antibacterial effect. The correlation coefficient (R^2^) is applied to evaluate the relationship between PK/PD parameters and antibacterial effect; the higher of the value of R^2^, the closer the PK/PD parameters are related to effect.

## MPC-Based PK/PD Integration

**Figure 2:**
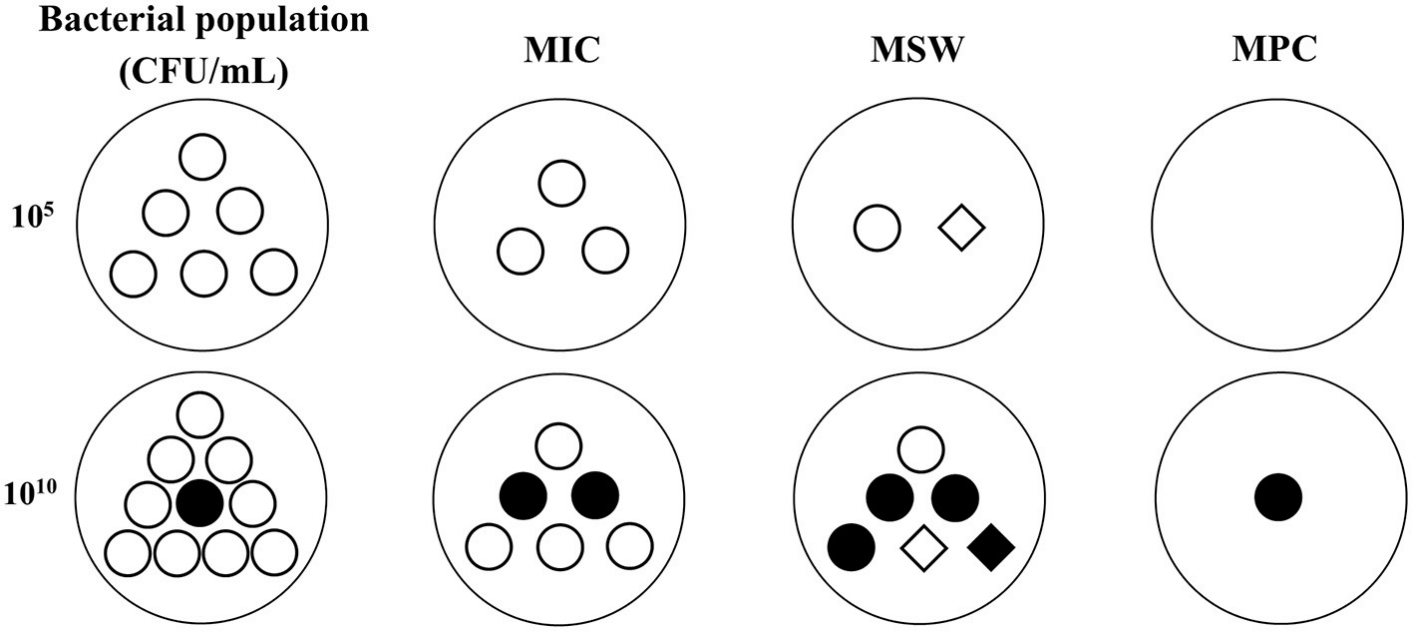
The possible changes of bacterial population and sensitivity under different antibacterial concentrations in low (10^5^ CFU/mL) and high (10^10^ CFU/mL) inoculums based on MSW theory. ◦, susceptible bacteria. •, natural mutant bacteria. ♢, first step mutant bacteria. ♦, multiple step mutant bacteria. MIC, minimum inhibitory concentration. MPC, mutant prevention concentration. MSW, mutant selection window, is the drug concentration between MIC and MPC. In low inoculum, the bacterial population will be gradually reduced with the drug concentrations added from MIC to MPC, and the bacterial sensitivity may be decreased because of the emergence of first step resistant mutation during MSW. In high inoculum, a natural mutant bacteria may exist in the initial population. The total population will be decreased but the mutant subpopulations may be increased when the drug concentrations below MPC. The sensitivity may be obviously decreased because of the emergence of first step and multiple step mutant bacteria during MSW.

To cope with the low bacterial cell density in MIC tests, some researchers have explored other PD parameters for serious infections (>10^10^ CFU/mL) for PK/PD integration. In serious infections, susceptible bacteria may produce a natural gene mutation (first-step mutant bacteria) that are resistant to drugs. At low drug concentrations, susceptible bacteria will gradually be killed and first step mutant bacteria may reproduce rapidly, which may result in recurrence of infection and further resistant mutations. Therefore, a new PD variable was proposed for high bacterial cell densities. MPC is the MIC of drugs for high bacterial cell densities (10^10^ CFU/mL) that can inhibit the growth of first step mutant bacteria (74, 75). In clinical infections, the bacterial cell density generally cannot exceed 10^12^ CFU/mL, hence two-step mutations rarely occur under natural conditions, but they may easily arise under low drug concentrations. Therefore, it is of great significance to prevent the emergence of drug-resistant bacteria when designing the dosage regimen based on MPC. Meanwhile, a mutant selection window (MSW) theory was proposed based on MIC and MPC (the corresponding PK/PD parameters are listed in Figure 1). This theory considers that drug-resistant bacteria are easily induced in the middle part of the MSW (the upper part cannot produce effect because the size of the bacterial population is large, and the lower part may have no effects because of the smaller bacterial population densities which have just been greatly reduced by previous concentrations; Figure 2). Therefore, a rational dosage regimen should be carefully designed that is not only an effective bacterial treatment, but that also prevents the emergence and spread of resistant bacteria.

Various studies have applied MPC as a PD variable (76–81). MPC-based PK/PD integration has mainly been applied for fluoroquinolones, because the mechanism of resistant mutations in the MSW theory is consistent with the mutant resistance mechanism of fluoroquinolones that usually occurs gradually through gene point mutations. Cui et al. (79) applied a rabbit TCIM to study the antibacterial activity of levofloxacin against *Staphylococcus aureus*, and analyzed the relationship between AUC_24h_/MPC and the generation of drug-resistant bacteria. The results showed that the emergence of resistant mutants could be inhibited when AUC_24h_/MPC >25 h. Liang et al. (81) applied three kinds of levofloxacin-resistant *S. aureus* strains (with the same MIC but different MPC) to compare the difference between AUC_24_/MIC and AUC_24_/MPC as PK/PD parameters for inhibiting the generation of drug-resistant bacteria. The results showed that generation of drug-resistant mutants could be inhibited when AUC_24_/MPC values were between 22 and 25 (even for strains with different MPC values). When applying AUC_24_/MIC as the target PK/PD parameter, the values were 8-fold different despite having the same MIC. Therefore, AUC_24_/MPC was more suitable than AUC_24_/MIC as the target PK/PD parameter to inhibit the generation of drug-resistant mutations. Zhang et al. (46) carried out a mutation window study on danofloxacin against *Actinobacillus pleuoniae* using a porcine TCIM, and analyzed the relationship between AUC_24h_/MPC and the generation of drug-resistant bacteria. The results showed that the generation of drug-resistant bacteria could be significantly inhibited when AUC_24h_/MPC >18.58 h. These studies shown that the susceptible or resistant mutant bacteria could be inhibited when the value of AUC/MIC or AUC/ MPC were 24 h which just means that the average plasma concentration over 24 h is equal to the MIC or the MPC.

In order to expand the application of the MSW approach, various types of antibacterials have been studied. For time-dependent drugs, %T> MIC is the PK/PD parameter that best reflects antibacterial activity. For MPC-based PK/PD parameters, %T >MPC was applied to prevent mutations, which reflects the time percentage of the drug concentration within the MSW during the interval of dosage administration. Alieva et al. (82) applied an *in vitro* dynamic model to study the relationship between the residence time (T_MSW_) of linezolone concentration within the MSW and the emergence of drug-resistant bacteria. The results showed that the correlation between T_MSW_ and T >MPC and area under the bacterial curve of mutation (AUBC_M_) was 0.99. Thus, T_MSW_ was an important indicator for predicting the emergence and enrichment of drug-resistant bacteria. Xiong et al. (48) applied a rabbit TCIM to study resistance mutations for cefquinome in *S. aureus*, and the results showed that drug-resistant bacteria appeared and accumulated when T >MIC_99_ >70% or T >MPC <58%. Zhang et al. (49) applied a pig TCIM to study the MSW of cefquinome against *Escherichia coli*, and the results showed that the emergence and enrichment of resistant bacteria occurred when T >MIC_99_ >25% or T >MPC <50%. In these studies, TCIM was used for dose regimens testing because this model do not allow extrapolation to dose regimens for clinical use, and the concentration time profiles within tissue cages do not mirror those of extracellular fluid because of the perfusion barriers.

Therefore, there was an optimal value to prevent the emergence and spread of drug-resistant bacteria when applying MPC-based PK/PD integration studies (83–87). However, the mechanism of drug-resistant bacterial resistance have a variety of ways. Such as except for genetic mutations, the emergence of resistant bacteria may be due to the spread of exogenous plasmid, especially for drugs other than fluoroquinolones. Therefore, the use of MSW theory is dispute which need more exploration.

## Multiple PD Parameter-Based PK/PD Integration

To investigate antibacterial activity in more detail, time-kill curves with multiple endpoints can be applied for PK/PD integration. Compared with PK/PD integration based on MIC alone, this multi-parameter-based time-kill curve model can provide detailed and accurate descriptions of dynamic changes in bacterial growth and death rates under different drug concentrations. Depending on whether drug concentrations change, this model can be divided into static and dynamic models. Static models are mainly conducted by comparing changes in the number of bacteria to obtain the bacterial growth rate constant and the death rate under different drug concentrations, and the corresponding equation is applied to analyze the relationship between drug concentration and antibacterial effect. However, in static models, the drug concentration is constant, whereas dynamic changes in drug concentration occur in the host. Dynamic models can be conducted by peristaltic pump and hollow fiber models to simulate dynamic changes in drugs and antibacterial effects in the clinic, and they may more accurately reflect clinical therapeutic effects.

Nolting et al. (88) studied the *in vitro* bactericidal effect of piperacillin against *E. coli* and applied a modified multi-parameter E_max_ model (Equation 2) to describe the bactericidal effect (dN/dt, change in bacteria over time). This model was used to investigate the maximum bactericidal effect (K_max_), the normal growth rate of bacteria (K_growth_), drug concentration (C_t_), the concentration that achieves a 50% maximum bactericidal effect (EC_50_), the delayed growth constant (Z), and the initial bacterial population (N). This model can dynamically describe changes in bacterial population over time under different drug concentrations. The results showed that the bactericidal effect of piperacillin was closely related to the time of drug administration; the more frequent the administration, the better the antibacterial effect. Compared with models base on MIC alone, this model can provide more detailed and accurate guidance for designing the dosage regimen.


(2) $$\begin{array}{l} \frac{dN}{dt}=\left(\frac{{\text{k}}_{\text{gowth}}-{\text{K}}_{\text{max}}\times {\text{C}}_{\text{t}}}{\text{E}{\text{C}}_{50}+{\text{C}}_{\text{t}}}\right)\times \left(1-{\text{e}}^{-\text{zt}}\right)\times \text{N} \end{array}$$

Regoes et al. (89) established a multiple PD parameter mathematical model (Equation 3) to study the PK/PD integration of five antibacterials against *E. coli*. In this model, ψ(a) is the net bacterial growth rate under different drug concentrations (a), ψ_max_ is the maximum bacterial growth rate in drug-free medium, ψ_min_ is the minimum bacterial net growth rate in drug-containing medium, N is the Hill coefficient (the slope of the graph of drug concentrations against bacterial net growth rate), and zMIC represent the MIC values of different bacteria. The results showed that when MIC was the same, the larger the value of k, the better the bactericidal effect, and the smaller the value of ψ_min_, the better the bactericidal effect. In this model, four PD parameters were employed, which can more precisely reflect the antibacterial characteristics of drugs to help design a more accurate and rational dosage regimen. Foerster et al. (90) also applied this model to study the antibacterial activities of several types of drugs against five strains of *Neisseria gonorrhoeae*.


(3) $$\begin{array}{l} \psi (a)=\psi_{\max}-\frac{\psi_{\max}-\psi_{\min}\times {\left(\frac{\text{a}}{\text{zMIC}}\right)}^{\text{N}}}{{\left(\frac{\text{a}}{\text{zMIC}}\right)}^{\text{N}}-\frac{\psi_{\min}}{\psi_{\max}}} \end{array}$$

This model also have been applied to classify bacteria as susceptible or resistant. Chauzy et al. (91) studied the antibacterial activity of polymyxin B against susceptible and drug-resistant *Klebsiella pneumoniae* using two consecutive bactericidal kill curve experiments and multiple PD parameter-based PK/PD integration. The results showed that after two consecutive bactericidal experiments, the bacteria whether appeared induced mutations could be determined by analyzing changes in growth and death rates.

In conclusion, compared PK/PD integration based on MIC alone, multiple PD parameter-based PK/PD models can more accurately reflect the antibacterial effects of drugs against pathogens, which has important implications for designing a rational dosage regimen to prevent the emergence of resistant mutant pathogens.

## PK/PD Integration Based on Kill Rate

**Figure 3:**
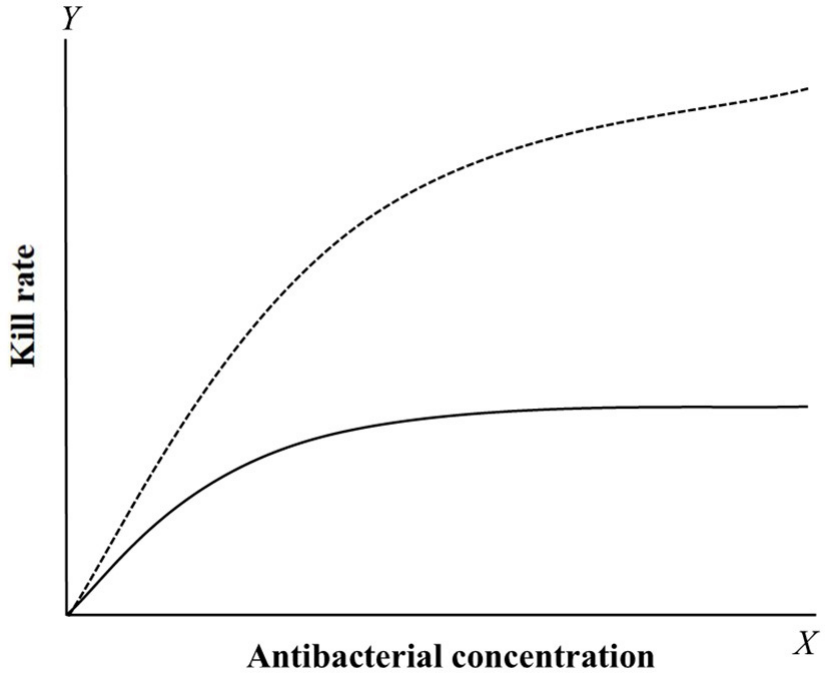
The relationship curves between kill rate and concentration of concentration-dependent (dotted line) and time-dependent (solid line) drugs. For concentration-dependent drugs, the kill rate will gradually increase with the concentration added. For time-dependent drugs, the kill rate was smaller compared to concentration-dependent drugs and rapidly reach a plateau that the value have little change with the concentration increased.

Kill rate is a PD reference that can be obtained from time-kill curves (92–98). It is the slope of time-kill curves, which reflects the interaction between bacterial growth and death rates under different drug concentrations. Compared with multiple PD parameter-based PK/PD integration, this model can directly analyze the relationship between kill rate and drug concentrations at different time periods. This method can dynamically analyze changes in antibacterial activity over time, and it has been used to classify antibacterial characteristics (concentration-dependent or time-dependent drugs). Two classical curves are depicted in Figure 3 to describe the relationship between kill rate and drug concentration. For time-dependent drugs, the value of kill rate increases with increasing drug concentration, but it is relatively small compared with concentration-dependent drugs at low concentrations. Furthermore, a maximum value is reached and it no longer increases with increasing drug concentration after this point. For concentration-dependent antibacterials, the value of kill rate increases quickly with increasing drug concentration. This antibacterial characteristic can be directly applied for drug classification.

Ferro et al. (99) analyzed the antibacterial activities of a variety of antibacterial drugs against two types of fast-growing *Mycobacterium* strains. For *M. abscess*, the maximum kill rate occurred between 24 and 72 h, and the maximum kill rate was 0.0427 h^−1^, 0.0231 h^−1^, and 0.0142 h^−1^ for amikacin, clarithromycin, and cefoxitin, respectively. For *M. fortuitum*, the maximum kill rate occurred between 3 and 24 h, and amikacin had the strongest antibacterial activity with a maximum bactericidal rate of 0.1933 h^−1^. Zhang et al. (100) analyzed the relationship between kill rate and drug concentration for doxycycline against *Mycoplasma gallisepticum* at different time periods, and the results showed that the optimal time period was 0-48 h (R^2^ = 0.986) and the maximum kill rate was 0.11^−1^ h. Zhang et al. (101) studied the kill rate of cefquinome against *A. pleuropneumoniae* and analyzed the relationship between kill rate and drug concentrations at different time periods. The results showed that cefquinome exerted time-dependent antibacterial activity, the optimal time period was 0-9 h (*R*^2^ = 0.9955), and the maximum kill rate was 0.48 log_10_ CFU/mL/h. Maneke et al. (102) compared the difference in kill rate between cephalexin and kanamycin alone and in combination against *E. coli, S. aureus, Streptococcus agalactiae, Streptococcus dysgalactiae*, and *Streptococcus uberis*. The results showed that the drug combination could increase the kill rate more than either drug alone, which can shorten the time required to achieve a bactericidal effect, and the antibacterial activity was concentration-dependent.

Compared with MIC-based PK/PD integration, the kill rate for based PK/PD integration can reflect dynamic changes in antibacterial activity, which can be widely used in the selection of new drugs, design of the dosage regimen, and monitoring changes in bacterial sensitivity.

## Conclusion

In conclusion, MIC based PK/PD is the most commonly applied method for evaluating antibacterial activities. However, with the emergence and spreading of resistant mutant bacteria, MIC-only-based PK/PD integration cannot meet the needs of clinical medicine due to the limitations of MIC. Therefore, it is necessary to optimize PD parameters for detailed analysis of antibacterial activity. Further research on MPC, multiple PD parameters, and kill rate-based PK/PD integration will gradually overcome the limitations of MIC, and these could assist optimizing the dosage regimen and prevent or slow the emergence of resistant mutations.

## Author Contributions

LZ, HX, YW, and HW contributed to the methodology, software use, validation, formal analysis, and preparation of the manuscript writing—reviewing and editing. GZ and JH contributed to the manuscript supervision. All authors contributed to the article and approved the submitted version.

## Funding

This work was supported by the Postdoctoral Research Foundation of Henan Province (202002067), Key Technology Research and Development Program of Henan Province (212102110373), the Scientific Research Program of Henan Institute of Science and Technology (103010620002/004), Postdoctoral Research Foundation of Henan Institute of Science and Technology, Program for Innovative Research Team (in Science and Technology) in University of Henan Province (22IRTSTHN026), and Key Scientific Research Projects of Universities in 2020 (20B230002).

## Conflict of Interest

The authors declare that the research was conducted in the absence of any commercial or financial relationships that could be construed as a potential conflict of interest.

## Publisher's Note

All claims expressed in this article are solely those of the authors and do not necessarily represent those of their affiliated organizations, or those of the publisher, the editors and the reviewers. Any product that may be evaluated in this article, or claim that may be made by its manufacturer, is not guaranteed or endorsed by the publisher.
